# The Alaris auditory evoked potential monitor as an indicator of seizure inducibility and duration during electroconvulsive therapy: an observational study

**DOI:** 10.1186/1471-2253-14-34

**Published:** 2014-05-13

**Authors:** Hsing-Hao Huang, Chun-Yu Wu, Feng-Sheng Lin, Yi-Ping Wang, Wei-Zen Sun, Chih-Peng Lin, Shou-Zen Fan

**Affiliations:** 1 Department of Anesthesiology, National Taiwan University Hospital, 7 Chung-Shan South Road, Taipei 10002, Taiwan; 2 Department of Pharmacology, College of Medicine, National Taiwan University, No. 1 Sec. 1, Jen-Ai Road, Taipei 100, Taiwan

**Keywords:** Auditory evoked potential, Electroconvulsive therapy, Seizure inducibility

## Abstract

**Background:**

Precise control of anesthetic depth during electroconvulsive therapy (ECT) is crucial because most intravenous anesthetics have anticonvulsant effects. In this study, we investigated the association between anesthetic depth measured by the Alaris auditory evoked potential index (AAI) and seizure inducibility and seizure duration during ECT.

**Methods:**

Sixty-four ECTs were evaluated in 12 consecutive patients. General anesthesia was performed with a thiopental-based method. The relationship between the pre-ictal AAI, seizure activity and seizure duration was analyzed, and a possible threshold pre-ictal AAI to induce a seizure duration of at least 25 seconds was calculated.

**Results:**

Forty-one of the 64 ECT stimuli successfully induced seizure activity that lasted longer than 25 seconds. Pre-ictal AAI was significantly correlated to seizure duration (r = 0.54, p < 0.001) and the threshold pre-ictal AAi value was calculated to be 26 (area under curve: 0.76, sensitivity: 70.3% and specificity: 73.9%, p < 0.001). ECT with a pre-ictal AAI ≧ 26 had a higher incidence of successful seizure activity ( p < 0.001) and a longer seizure duration (55 ± 35 v.s. 21 ± 27 seconds, p < 0.001).

**Conclusion:**

Maintenance of a pre-ictal AAI value ≧ 26 was associated with an increased incidence of successful seizure activities and a longer seizure duration. This is the first report to investigate Alaris AEP monitoring during ECT.

## Background

Electroconvulsive therapy (ECT) has gained importance over the years as a treatment for major affective disorders. This is partly due to the use of general anesthesia to reduce ECT-associated physical and psychological trauma [1]. An induced seizure is necessary during ECT, and a seizure duration more than 25 seconds is often required for successful treatment [2]. However, most intravenous anesthetics have negative effects on seizure duration due to their anticonvulsant effect. To optimize the potency of the ECT, a balance between adequate anesthesia and optimal duration of induced seizure is crucial. However, there is still a lack of consensus for a targeted level of anesthesia to maintain during the ECT.

Modern electroencephalogram (EEG)-based monitors such as bispectral index (BIS) and auditory evoked potential (AEP) allow a numeric description of the hypnotic component of anesthesia that by its instant availability enables the anesthesiologist to control anesthetic depth. Several reports have indicated a potential value of use of the BIS during the ECT [3-5]. In comparison, the potential role of AEP during ECT and the possible threshold AEP value needed to induce a successful seizure have not yet been clarified.

In the current study, we attempted to investigate the association between pre-ictal AEP score, incidence of seizure activity and seizure duration. In addition, we aimed to determine the possible threshold pre-ictal AEP score needed in order to induce successful seizure activity of more than 25 seconds.

## Methods

This observational study was approved by the Research Ethics Committee of National Taiwan University Hospital. After explanation of the protocol, written informed consent was obtained from each patient. ECT was then prescribed to a consecutive 12 adult patients experiencing endogenous depression or schizophrenia. Each patient had not receive ECT before. Otherwise eligible patients who had with cardiovascular or cerebrovascular comorbidities or drug allergies were excluded. All patients were treated with ECT more than five times (three times per week at 2-day intervals).

The Alaris AEP monitor is a commercially available AEP monitor designed to estimate depth of anesthesia. It generates an "Alaris AEP index" (AAI), which is a dimensionless number scaled from 100 (awake) to 0. An AAI of about 60–100 represents awake status. Before induction of general anesthesia, the Alaris AEP monitor electrodes (AEP electrodes; Danmeter A/S), and a headphone for auditory stimuli were applied as recommended by the manufacturers. The three AEP electrodes were placed at mid forehead, left forehead (reference) and left mastoid. Pre-ictal AAI values were obtained in each ECT treatment and the data was analyzed later by an independent individual. General anesthesia was induced with thiopental (3–6 mg/kg), with the dosage and timing determined by an anesthesiologist unaware of the purpose of the study and blinded to the AAI score. Patient's airway is supported by facilitated face mask ventilation by attending anesthesiologist and a laryngeal mask is readily available. The electroshock stimulus was delivered by a trained psychiatrist using an ECT stimulator (Mecta spECTrum 5000Q, Mecta Corp, Lake Oswego, Ore). The electrical current was applied bilaterally for 1–4 seconds with a fixed pulse width (1.0 ms), a fixed pulse frequency (90 Hz) and a fixed pulse current (800 mAmp). A maximum number of shocks for each treatment is three times. Duration of each electroshock was adjusted in a stepwise method which began from 0.5 second. If there was no no seizure activity with the duration longer than 25 seconds and then the shock duration would be escalated to 0.75 second or 1.0 second for the second or third electroshock respectively. The efficacy of the electrical stimulation was determined by the so-called tourniquet technique—that is, the seizure activity was observed by convulsive movements of the distal leg, around which an inflated tourniquet was set to block the entry of the depolarizing muscle relaxant (succinylcholine, intravenous bolus 0.5-1 mg/kg) [6]. The duration of each induced seizure was determined by monitoring the electroencephalogram seizure pattern and recorded. At the conclusion of the treatment, the patients were transferred to the recovery area after they had gained spontaneous breathing and could respond to verbal commands.

Those who performed data analysis was blinded to the treatment groups. The relationship between pre-ictal AAI and seizure duration was analyzed by the Pearson correlation coefficient test, and the threshold AAI needed o induce a successful seizure activity was determined by receiver operator characteristics curves (ROC), in which the area under the curve (AUC) was calculated. Wilcoxon rank-sum tests were applied to compare the pre-ictal AAI of successful or failed ECTs as well as the seizure durations of patients with a pre-ictal AAI above or below threshold value. The Fisher exact test was used to compare the incidence of successful seizure activity. The hypothesis was tested two-tailed at the p < 0.05 significance level with power > 0.9. All statistical analysis and graphs were performed using SigmaPlot for Windows, version 12 (SAS Institute, Cary, NC, USA).

## Results

Table 1 shows patient characteristics. Sixty-four ECT stimuli in 12 consecutive patients (age, 27–50 yr; six males and six females) were administered without any adverse events. None of these patient met the exclusion criteria and needed to be excluded. The diagnoses of the 12 patients enrolled were schizophrenia (N = 6), major depression (N = 3) and bipolar affective disorder (N = 3). Eight patients received a benzodiazepine-type hypnotic regularly, three patients received antidepressant regularly, and all patients received antipsychotic regularly.

**Table 1:** Patient characteristics

**Male/female (n)**	6/6
**Diagnosis (n)**	12
Schizophrenia	6
Major depression	3
Bipolar affective disorder	3
**Body weight (Kg)**	64.8 ± 9.2
**Height (cm)**	165.8 ± 8.6
**Seizure duration (second)**	39.6 ± 36.0
**Successful/failed ECTs**	41/23
**Psychotropic medication (n, %)**	
**Benzodiazepine type hypnotic**	8 (66.67%)
**Antidepressant**	3 (25%)
**Antiphychotic**	12 (100%)

ECT = electroconvulsive therapy.

Data are mean (±SD) and number (proportion, %).

The average time for ECTs was 4.3 ± 2.0 minutes. The average thiopental dosage was 3.9 ± 0.94 mg/kg. Twenty-three of the 64 ECT stimuli failed to induce successful seizure activity, and the other 41 ECTs all induced a successful seizure activity longer than 25 seconds.

All patients were unable to respond to verbal command and lost the eyelash reflex within 1 min after injection of thiopental. No patient could recall the ECT procedures during the follow-up examination in the general ward.

### The associations between pre-ictal AAI, seizure activity and seizure duration

**Figure 1:**
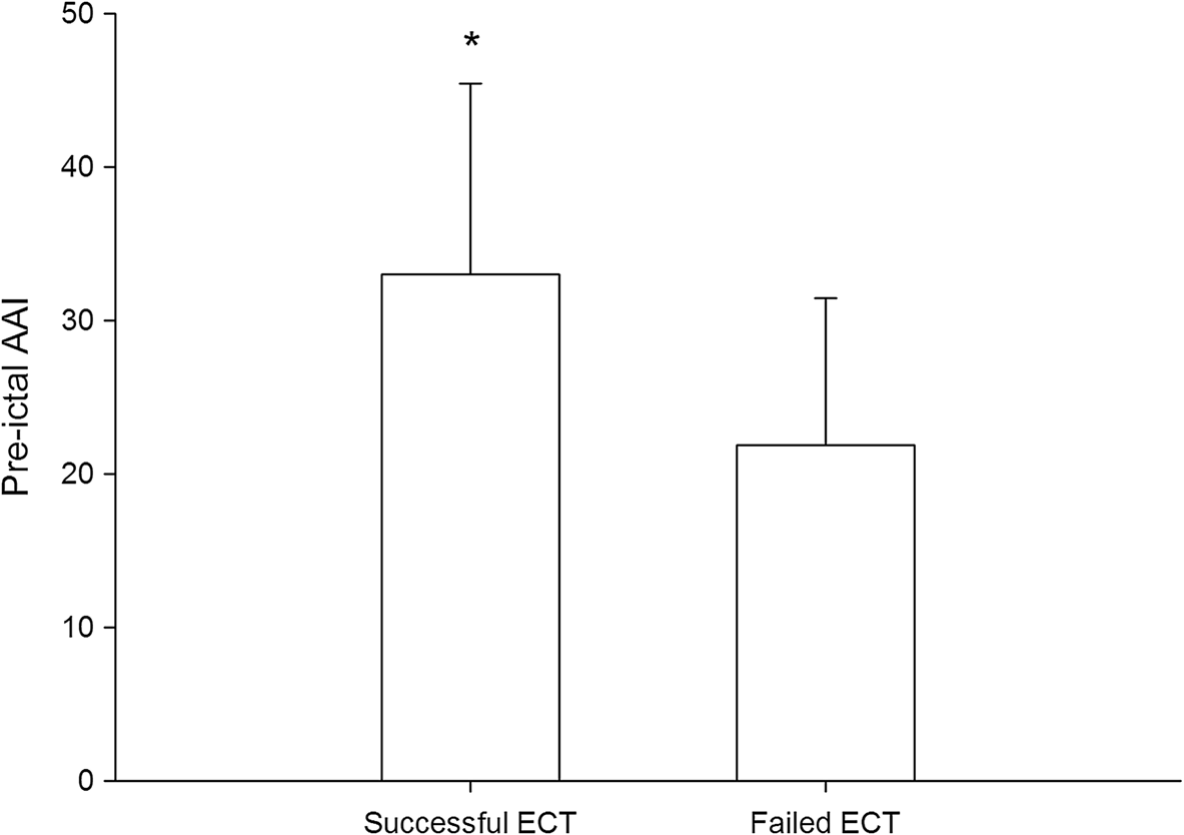
**Comparison of pre-ictal Alaris auditory evoke potential index (AAI) between successful and failed electroconvulsive therapy (ECT).** *means a significant difference with p < 0.05.

The mean pre-ictal AAI of the 64 ECT stimuli was 29 ± 13. The seizure duration of the 41 successful ECT stimuli was 39.6 ± 36.0 seconds. Pre-ictal AAI was mildly significantly correlated to the seizure duration (r^2^ = 0.29, p < 0.001). The pre-ictal AAI of failed 23 ECTs was significantly lower than that of successful 41 ECTs ( 22 ± 10 vs. 33 ± 12; p < 0.001, Figure 1). Administrated thiopental dosage was not correlated to seizure duration (p = 0.186). and the dosages used for failed and successful ECTs were not significantly different (p = 0.961).

**Figure 2:**
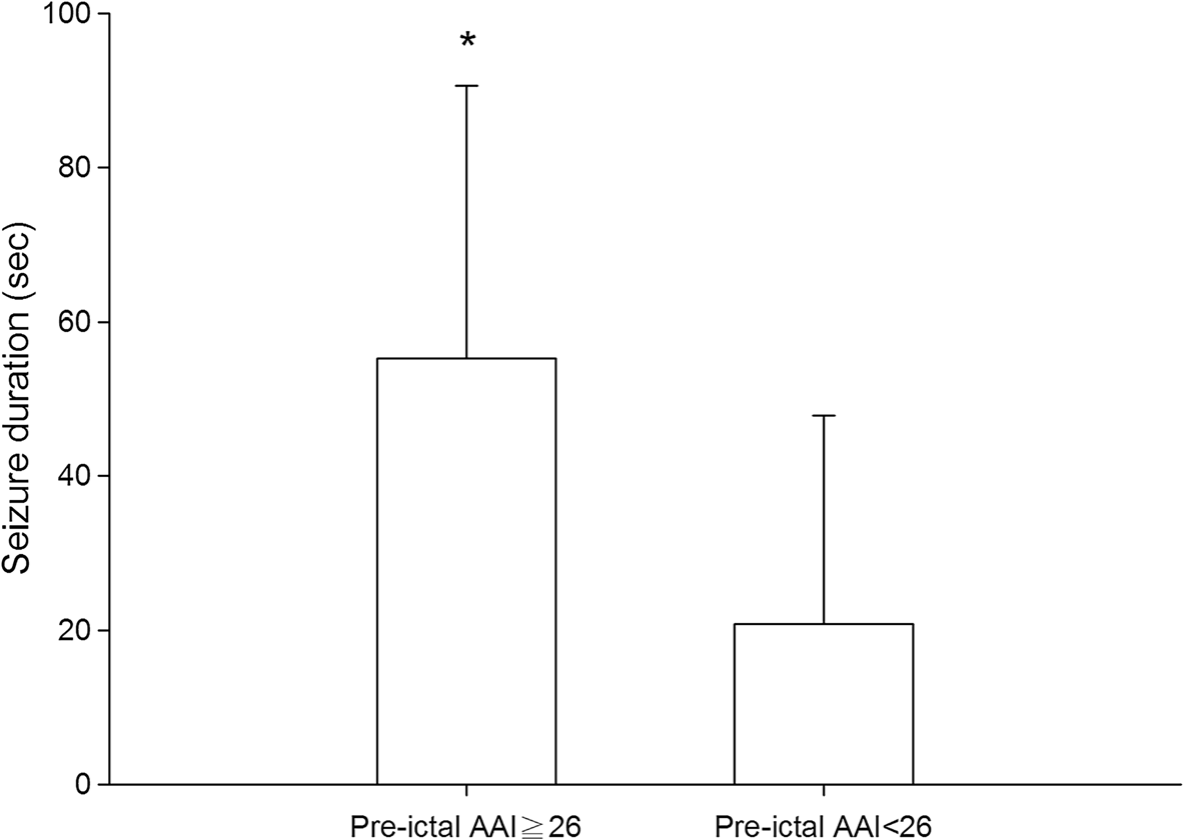
**Comparison of induced seizure duration between patients with a pre-ictal Alaris auditory evoke potential index (AAI) above or below 26.** *means a significant difference with p < 0.05.

The ROC curve derived for seizure duration more than 25 seconds indicated a threshold pre-ictal AAI of 26 (AUC: 0.76, p < 0.001; sensitivity of 71%, specificity of 74%; positive predictive value of 34% and negative predictive value of 93%). In patients with a pre-ictal AAI ≧ 26, there is more successful ECTs than in patients with a pre-ictal AAI < 26 (82.9% versus 27.6%, p < 0.001). The seizure duration in patients with a pre-ictal AAI ≧ 26 was 55 ± 35 seconds which was significantly longer than the seizure duration in those with a pre-ictal AAI < 26 of 21 ± 27 seconds (p < 0.001, Figure 2).

## Discussion

The current study has shown that pre-ictal AAI is not only associated with seizure activity but also with seizure duration, and that a pre-ictal AAI of at least 26 may be a threshold for adequate anesthesia and successful seizure induction during the ECT. To our best knowledge, this is the first study to investigate the performance of the Alaris AEP monitor during ECT.

During ECT, it is not appropriate to merely administer intravenous anesthetics using a set dosage range, because the patients are usually receiving various chronic psychoactive drugs that may interfere with seizure inducibility [1]. Because of this pre-existing variation in CNS depression, the intravenous anesthetic dosage may not be necessarily higher in the failed ECTs. This may be the reason that thiopental dosage was not significantly different between successful and failed ECTs in our study. Also, EEG-based monitors may have an important role as an overall gauge of CNS depression before ECT. Our study revealed a possible role of pre-ictal AAI in determining anesthetic depth appropriate for ECT, because the pre-ictal AAI was higher in the successful ECTs. Besides, by a high negative predictive property, 93% of shocks in patients with a pre-ictal AAI < 26 failed to induce a successful seizure activity. Therefore, a low pre-ictal AAI <26 should be avoided and so pre-ictal AAI may be a useful monitor to guide a more precise treatment. The efficacy of ECTs was previously believed to be related to a minimum seizure duration of 25 seconds and is currently believed to more related to occurrence of seizure [7]. Among patients with a pre-ictal AAI less than 26, the incidence of successful seizure activity was lower and the mean seizure duration (about 21 seconds) was shorter than recommended. In comparison, among patients with a pre-ictal AAI above 26, there was a high incidence of successful seizure activities, and the mean seizure duration was far longer than the recommended length (about 55 seconds). The pre-ictal AAI above 26 was also within a safe range of that previously reported for unconsciousness which was reported about 35 in both adult and pediatric surgical patients [8,9].

Both AAI and BIS can be considered effective measurements of anesthetic depth [10]. Most studies evaluating anesthetic depth monitoring were performed in the context of longer procedures. In comparison, an episode of ECT has a duration not more than several minutes long, so more rapidly responsive monitoring is required. Because of the average time delay of BIS is more than 30 seconds [11], this delay may limit the usefulness of BIS during ECT. In contrast to BIS, the AAI monitor allows extraction of the AEP signal within 15 to 25 sweeps of 110-msec duration; a process that results in only a 6-second delay [12]. Therefore, the AAI monitor may be an useful tool to determine a more precise timing for the electric shock of ECT. It has also reported that the BIS value that represents the awake state might become changed after the initial ECT [13]. In comparison, the AEP may be more resistant to the electroshock wave [14,15]. However, such characteristics of BIS and AEP during ECT need further investigation to confirm their validity.

There are several limitations in our study. First, because no previous report investigating AAI during ECTs, a convenience sampling is arranged in this observational study. The patient number is also small because that this population is relatively difficult to enroll. Therefore, the real value of usage of pre-ictal AAI during ECT may only be ensured by a randomized-controlled study, and our study may be a potential reference for further studies. Second, because methohexital is not available in our country, we used thiopental for ECT in our institute. Because different intravenous anesthetics have different proconvulsant and anticonvulsant effects [1], the threshold pre-ictal AAI value determined in this study may not be applicable to other intravenous anesthetics.

## Conclusions

In conclusion, this is the first report to investigate Alaris AEP monitoring during ECT. To maintain a pre-ictal value at least 26 was associated with an increased incidence of successful seizure activity and a longer seizure duration.

## Competing interests

The authors declare that they have no competing interests.

## Authors’ contributions

HHH participated in the study design and manuscript writing. CYW participated in the study design and manuscript writing. FSL participated in patient enrolment. YPW carried out the data collection. WZS carried out data analysis. CPL participated in the study design and manuscript writing. SZF audited study progress and helped to revise this manuscript. No conflicts of interest declared and no external fund obtained. All authors read and approved the final manuscript.

## Pre-publication history

The pre-publication history for this paper can be accessed here:

http://www.biomedcentral.com/1471-2253/14/34/prepub
